# Identification of New Metabolites of Bacterial Transformation of Indole by Gas Chromatography-Mass Spectrometry and High Performance Liquid Chromatography

**DOI:** 10.1155/2014/239641

**Published:** 2014-12-04

**Authors:** Pankaj Kumar Arora, Hanhong Bae

**Affiliations:** School of Biotechnology, Yeungnam University, Gyeongsan 712-749, Republic of Korea

## Abstract

*Arthrobacter* sp. SPG transformed indole completely in the presence of an additional carbon source. High performance liquid chromatography and gas chromatography-mass spectrometry detected indole-3-acetic acid, indole-3-glyoxylic acid, and indole-3-aldehyde as biotransformation products. This is the first report of the formation of indole-3-acetic acid, indole-3-glyoxylic acid, and indole-3-aldehyde from indole by any bacterium.

## 1. Introduction

Indole is a nitrogen-containing heterocyclic aromatic compound that has been considered as an environmental pollutant because of its worldwide occurrence in the environment [1]. It is highly toxic to human beings and may cause acute pulmonary edema, emphysema, hemoglobinuria, and hemolysis [2]. The exposure of plants to indole causes low pigmentation because of inhibition of biosynthesis of anthraquinone [3]. Indole has also been identified as an intercellular signal molecule in bacterial communities, controlling various bacterial physiological processes including spore formation, plasmid stability, drug resistance, biofilm formation, and virulence in indole-producing bacteria [4].

Many bacteria have the ability to degrade or transform indole and several pathways have been suggested for bacterial degradation of indole [5–10]. Sakamoto et al. [5] suggested degradation of indole via formation of indoxyl, 2,3-dihydroxyindole, isatin, N-formylanthranilate, anthranilate, salicylate, and catechol in a gram-negative bacterium isolated from tap water. Fujioka and Wada [6] studied the indole degradation pathway in a gram-positive coccus that utilized indole as its sole source of carbon and nitrogen and degraded it via 2,3-dihydroxyindole and anthranilate. Claus and Kutzner [7] isolated an indole mineralizing bacterium,* Alcaligenes* sp., from the activated sludge that degraded indole via indoxyl, isatin, anthranilic acid, and gentisate. Indole degradation has also been investigated under methanogenic conditions [8]. Wang et al. [8] studied anaerobic degradation of indole by a consortium of methanogenic bacteria that degraded indole to methane within seven to eighteen weeks. Berry et al. [9] reported transformation of indole to oxindole under methanogenic conditions. Madsen and Bollag [10] studied the indole degradation by a denitrifying microbial community and identified oxindole, isatin, and anthranilic acid as metabolites.

Bacterial transformation of indole into various compounds has also been studied [2, 11]. Shi et al. [11] reported transformation of indole into indigo by* Escherichia coli* cells expressing phenol hydroxylase. Qu et al. [2] reported the cells of* Escherichia coli* expressing biphenyl dioxygenase and biphenyl-2,3-dihydrodiol-2,3-dehydrogenase of* Dyella ginsengisoli* LA-4 transformed indole to indigo, indirubin, and isatin. Indigo was the major transformation product. In this communication, we report novel transformation of indole into a few indole derivatives by the previously isolated bacterium,* Arthrobacter* sp. SPG.

## 2. Materials and Methods

### 2.1. Chemicals

Indole, indole-3-acetic acid, and other indole derivatives were purchased from Sigma-Aldrich. All other chemicals, reagents, and solvents were purchased from Fisher Scientific.

### 2.2. Bacterial Growth and Indole Transformation

*Arthrobacter* sp. SPG, which was isolated previously from a pesticide contaminated site, India, and utilized 4-nitrophenol as its sole carbon and energy source, was used for this study [12]. Strain SPG was grown in a 500 mL Erlenmeyer flask containing 200 mL minimal medium, 0.5 mM indole, and 10 mM sodium succinate, and the flask was incubated at 30°C under shaking (200 rpm). The minimal medium contained Na_2_HPO_4_ (4 g/L), KH_2_PO_4_ (2 g/L), MgSO_4_7H_2_O (0.8 g/L), and (NH_4_)_2_SO_4_ (0.8 g/L), 1 mL trace element solution [12]. The trace element solution was prepared as described previously [12]. Samples were collected at regular intervals (0 h, 4 h, 8 h, 12 h, 16 h, 20 h, 24 h, 28 h, 32 h, 36 h, and 40 h) and analyzed for growth and indole depletion. The growth of strain SPG was measured spectrophotometrically taking the optical density at 600 nm, and depletion of indole was measured by high performance liquid chromatography (HPLC).

HPLC analysis of the samples was carried out using a Waters 600 HPLC model equipped with a photodiode array detector. The metabolites were separated on a C-18 column using a linear gradient (solvent A; 1% acetic acid, solvent B; 100% acetonitrile) with a flow rate of 1.5 mL/min. Injection volume was 20 *μ*L and metabolites were detected at 280 nm.

To monitor the indole transformation and identify the metabolites of indole transformation, the samples collected at regular intervals were centrifuged and extracted with ethyl acetate. The extracted samples were analyzed by high performance liquid chromatography (HPLC) and gas chromatography-mass spectrometry (GC-MS).

An Agilent gas chromatography system with a high throughput time-of-flight mass spectrometer was used with a column HP-5 (30 m × 0.320 mm × 0.25 *μ*m). The column temperature was initially increased from 50°C to 280°C at the rate of 20°C/min and then held for 5 min. Helium was used as a carrier gas at 1.5 mL/min and the samples (1 *μ*L) were injected in splitless mode. The ion-source temperature and transfer line temperature were maintained at 250°C and 225°C, respectively. The electron energy was set at 70 eV.

## 3. Results and Discussion

**Figure 1:**
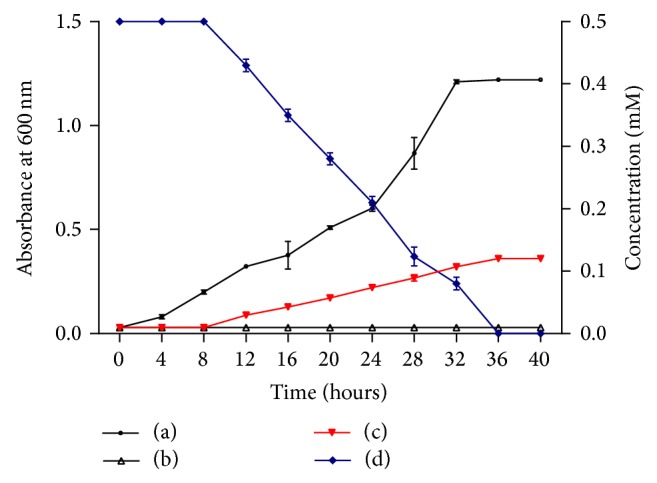
Growth of* Arthrobacter* sp. SPG in (a) minimal medium containing 10 mM sodium succinate, (b) minimal medium containing 0.5 mM indole, and (c) minimal medium containing both 10 mM sodium succinate and 0.5 mM indole. (d) Indole depletion by* Arthrobacter* sp. SPG.

*Arthrobacter* sp. SPG was able to transform 0.5 mM indole in the presence of sodium succinate. The growth of strain SPG was measured in minimal medium containing 10 mM sodium succinate and 0.5 mM indole. Strain SPG grew well in minimal medium containing 10 mM sodium succinate as its sole source of carbon and energy. However, there was no bacterial growth on minimal medium containing 0.5 mM indole as its sole source of carbon and energy. The bacterial growth was significantly reduced in medium containing 10 mM sodium succinate and 0.5 mM indole and the maximum optical density of the culture was 0.35 after 40 hours of incubation (Figure 1). Indole depletion was measured by HPLC and it was observed that indole was completely depleted/transformed within 36 h.

**Figure 2:**
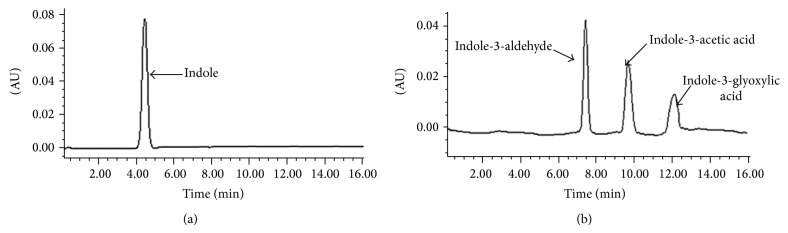
HPLC analysis of samples of indole biotransformation by* Arthrobacter* sp. SPG. (a) 8 h sample showing only peak for indole and (b) 36 h sample showing complete bacterial transformation of indole into indole-3-acetic acid, indole-3-glyoxylic acid, and indole-3-aldehyde.

HPLC analysis also showed the complete transformation of indole into three metabolites (Figure 2). In the sample of 8 h, only peak of indole was detected, whereas in the sample of 36 h, indole was completely depleted with appearance of three metabolites. These metabolites were identified as indole-3-acetic acid, indole-3-glyoxylic acid, and indole-3-aldehyde on the basis of the comparison of their retention times with those of authentic standards. The retention times of these three metabolites were 9.60, 12.0, and 7.48 min, respectively, whereas retention time of the indole was 4.25 min.

**Figure 3:**
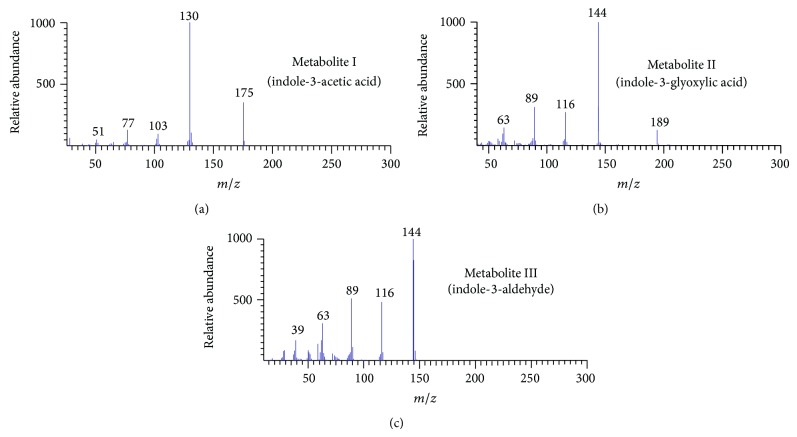
GC-MS analysis showing mass spectrum of metabolite I (indole-3-acetic acid, (a)), metabolite II (indole-3-glyoxylic acid, (b)), and metabolite III (indole-3-aldehyde, (c)).

GC-MS analysis showed that the mass fragment patterns of metabolites I, II, and III corresponded to those of authentic standards of indole-3-acetic acid, indole-3-glyoxylic acid, and indole-3-aldehyde, respectively (Figure 3). The mass spectrum of metabolite I had molecular ion at* m/z* 175 and quinolinium ion at* m/z* 130 (Figure 3(a)). The mass spectrum of metabolite II showed a molecular ion peak at* m/z* 189 and other fragments were observed at* m/z* values of 144, 116, 89, and 63 (Figure 3(b)). The mass spectrum of metabolite III showed a molecular ion at* m/z* 144 and the major fragments were observed at* m/z* values of 116, 89, and 63 (Figure 3(c)). The results of GC-MS confirmed the identities of metabolites I, II, and III as indole-3-acetic acid, indole-3-glyoxylic acid, and indole-3-aldehyde, respectively.

Literature studies indicate that indole-3-acetic acid has previously been identified as a catabolic product of tryptophan metabolism [13, 14]. L-Tryptophan metabolism most commonly occurred in microorganisms through the indole-3-pyruvic acid pathway, in which tryptophan is first converted to indole-3-pyruvic acid by tryptophan aminotransferase [13]. Indole-3-pyruvic acid was then decarboxylated to indole-3-acetaldehyde by an indole-3-pyruvic acid decarboxylase, which was further oxidized to indole-3-acetic acid by aldehyde dehydrogenase [13]. Tryptophan may also be converted to indole-3-acetic acid via the indole-3-acetamide pathway [14]. Initially, tryptophan-2-monooxygenase converted tryptophan to indole-3-acetamide which was further converted to indole-3-acetic acid by indole-3-acetamide hydrolase [14]. Forni et al. [15] reported that cells of* Arthrobacter* species produced indole-3-acetic acid in the culture medium when precursor tryptophan was present in the medium. No indole-3-acetic acid production was observed in the absence of tryptophan [15]. Idris et al. [16] reported tryptophan-dependent production of indole-3-acetic acid by the plant-beneficial rhizobacterium* Bacillus amyloliquefaciens* FZB42. The production of indole-3-acetic acid was observed in 80% of bacteria isolated from the rhizosphere; however, there is limited information about the production of indole-3-acetic acid by gram-positive free-living soil bacteria. A gram-positive phytopathogen* Rhodococcus fascians* also synthesized indole-3-acetic acid via the indole-3-pyruvic acid pathway [17]. The formation of indole-3-acetic acid in this phytopathogen was associated with developmental alterations, such as leafy galls, on a wide range of plants [17].

The tryptophan-independent production of indole-3-acetic acid is also known in the literature [18, 19]. Prinsen et al. [18] reported indole-3-acetic acid biosynthesis in* Azospirillum brasilense* by a tryptophan-independent pathway. In our study, when strain SPG was grown on tryptophan-supplemented minimal medium containing sodium succinate, we did not observe indole-3-acetic acid or any other indole derivatives in the medium. These data suggest that the formation of indole-3-acetic acid and related derivatives is independent of tryptophan metabolism in this study.

**Figure 4:**
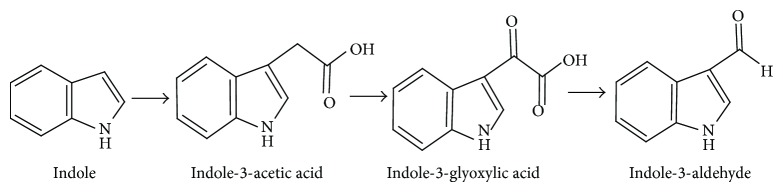
Proposed pathway of indole transformation for* Arthrobacter* sp. SPG.

Indole-3-aldehyde and indole-3-glyoxylic acid have been identified as the products of the enzymatic oxidation of indole-3-acetic acid [20]. Stutz [20] also reported the enzymatic formation of indole-3-aldehyde from indole-3-acetic acid via indole-3-glyoxylic acid. We have also detected indole-3-aldehyde and indole-3-glyoxylic acid, which could be formed from indole-3-acetic acid in the indole biotransformation pathway. On the basis of the above discussion, we propose a tryptophan-independent pathway for indole biotransformation. Indole was first converted to indole-3-acetic acid via a tryptophan-independent pathway, which was further oxidized to indole-3-glyoxylic acid and then to indole-3-aldehyde (Figure 4).

In this study, indole was degraded via formation of indole-3-acetic acid which is a plant growth promoting compound, controlling many important physiological processes including cell enlargement and division, tissue differentiation, and responses to light and gravity [21]. Bacterial degradation of indole-3-acetic acid has been studied by several researchers [22–25]. Leveau and Lindow [22] studied catabolism of indole-3-acetic acid by* Pseudomonas putida *strain 1290 that degraded indole-3-acetic acid via formation of catechol. Proctor [23] showed that* Pseudomonas* strain degraded indole-3-acetic acid via skatole (3-methyl indole), indoxyl (3-hydroxy indole), salicylic acid, and catechol. Egebo et al. [24] suggested that* Bradyrhizobium japonicum* degrades indole-3-acetic acid via* o*-formaminobenzoylacetic acid,* o*-aminobenzoylacetic acid, and anthranilic acid. Jensen et al. [25] reported that* Bradyrhizobium japonicum* may degrade indole-3-acetic acid via dioxindole-3-acetic acid, dioxindole, isatin, *α*-aminophenyl glyoxylic acid (isatinic acid), and anthranilic acid. In our studies, indole-3-acetic acid is converted to indole-3-glyoxylic acid and then to indole-3-aldehyde.

The present study of indole biotransformation is different from all the previous studies since we have detected new metabolites previously not found [5–7]. Previous studies showed that indole biodegradation occurred via the formation of (i) indoxyl, 2,3-dihydroxyindole, isatin, N-formylanthranilate, anthranilate, salicylate, and catechol [5]; (ii) 2,3-dihydroxyindole and anthranilate [6]; (iii) indoxyl, isatin, anthranilic acid, and gentisate [7]; and (iv) indigo [11]. None of these previously reported metabolites was formed during the transformation of indole by strain SPG.

*Arthrobacter* sp. SPG exhibits great potential to degrade various nitrogen-containing aromatic compounds including 4-nitrophenol (4NP), 2-chloro-4-aminophenol (2C4AP), 2-chloro-4-nitrophenol, 2-nitrobenzoate, 3-methyl-4-nitrophenol, nitrocatechol, and indole [12]. Detailed studies have been carried out on the degradation of 4NP, 2C4AP, and indole [12, 26]. Strain SPG utilized 4NP and 2C4AP as its sole sources of carbon and energy and degraded them via the hydroquinone pathway [12, 26]. However, strain SPG did not utilize indole as its sole source of carbon and energy but transformed it completely into indole-3-acetic acid, indole-3-glyoxylic acid, and indole-3-aldehyde. In future, the metabolic potential of strain SPG may be further explored using the approaches of genomics and proteomics.

## 4. Conclusion

This study clearly showed that* Arthrobacter* sp. SPG biotransformed indole into indole-3-acetic acid, indole-3-glyoxylic acid, and indole-3-aldehyde. This is the first report of the formation of indole-3-acetic acid, indole-3-glyoxylic acid, and indole-3-aldehyde from indole by any bacterium.
